# Glutathione Decrement Drives Thermogenic Program In Adipose Cells

**DOI:** 10.1038/srep13091

**Published:** 2015-08-11

**Authors:** Daniele Lettieri Barbato, Giuseppe Tatulli, Stefano Maria Cannata, Sergio Bernardini, Katia Aquilano, Maria R. Ciriolo

**Affiliations:** 1 Dept. Biology, University of Rome “Tor Vergata”, Via della Ricerca Scientifica 1, 00133 Rome, Italy; 2 Scientific Institute for Research Hospitalization and Health Care and Università Telematica San Raffaele Roma, Via di Val Cannuta 247, 00166 Rome, Italy

## Abstract

Adipose tissue metabolically adapts to external stimuli. We demonstrate that the induction of the thermogenic program in white adipocytes, through cold exposure in mice or *in vitro* adrenergic stimulation, is accompanied by a decrease in the intracellular content of glutathione (GSH). Moreover, the treatment with a GSH depleting agent, buthionine sulfoximine (BSO), recapitulates the effect of cold exposure resulting in the induction of thermogenic program. In particular, BSO treatment leads to enhanced uncoupling respiration as demonstrated by increased expression of thermogenic genes (e.g. Ucp1, Ppargc1a), augmented oxygen consumption and decreased mitochondrial transmembrane potential. Buffering GSH decrement by pre-treatment with GSH ester prevents the up-regulation of typical markers of uncoupling respiration. We demonstrate that FoxO1 activation is responsible for the conversion of white adipocytes into a brown phenotype as the “browning” effects of BSO are completely abrogated in cells down-regulating FoxO1. In mice, the BSO-mediated up-regulation of uncoupling genes results in weight loss that is at least in part ascribed to adipose tissue mass reduction. The induction of thermogenic program has been largely proposed to counteract obesity-related diseases. Based on these findings, we propose GSH as a novel therapeutic target to increase energy expenditure in adipocytes.

Adipose tissue has multiple metabolic functions and is primary involved in the regulation of energetic homeostasis. The traditional nomenclature has featured adipose tissue as white and brown depending on its macroscopic appearance. White adipose tissue (WAT) is constituted of white adipocytes and has generally been viewed as an energy reservoir of neutral lipids. WAT stores the excess of calories for their usage by other tissues during nutrient scarcity. Brown adipose tissue (BAT) primarily represents the site of thermoregulation. Actually, BAT is an oxidative tissue that, when fully activated, uses fatty acids stored into brown adipocytes to support the activity of the uncoupling protein 1 (Ucp1 or thermogenin) that dissipates the mitochondrial proton gradient to produce heat.

WAT is capable of great plasticity and undergoes a continuous shape remodeling during life in response to many environmental stimuli (drugs, nutrients, etc) and physiopathological conditions (e.g. ageing, obesity)^1^,^2^,^3^. An unexpected presence of cells with the characteristic of brown adipocytes was noted in WAT^4^,^5^. Hence, the classification of adipocytes has been recently updated. In particular, brite (brown-in-white) or beige adipocytes is the name used to distinguish brown-like cells located into WAT from white and brown adipocytes^6^,^7^. Importantly, upon physiological stimuli, such as physical exercise and cold exposure, WAT can evolve towards a brown-like phenotype^8^,^9^,^10^,^11^. During this process (browning) the expression of classic BAT genes is triggered in WAT, thus increasing energy expenditure and leading to slimming. Consequently, WAT browning positively impacts on susceptibility to obesity adiposity, insulin resistance and hyperlipidaemia^2^,^12^. However, also pathological conditions, such as inflammation and cancer are causative of WAT browning^13^,^14^,^15^.

There is also a large new consensus that metabolically active BAT is present in humans from newborns to adults^16^, and lower amounts of BAT are associated with obesity and diabetes^17^,^18^. This has renewed the interest towards BAT function and the effects of its recruitment on overall body energy metabolism. The increase in energy expenditure through WAT browning or BAT activation is therefore an attractive goal to reduce the risk of obesity and its-related pathologies^17^,^18^.

An intricate pathway of gene expression governs both the development of an adipocyte phenotype and metabolic response to specific inputs. It is recently emerging that the induction of genes that orchestrate adipose cell metabolism and differentiation is driven by changes in intracellular redox state^19^,^20^. Moreover, an oxidative redox potential seems to modulate the efficiency of mitochondrial metabolism^21^. In this regard, mitochondria of brown adipocytes have higher level of reactive oxygen species (ROS) production and more oxidized status when compared to other cell types^21^. Exposure to cold further shifts mitochondria redox status of brown adipocytes towards pro-oxidant condition and this event is required for the proper functioning of BAT mitochondria^21^. These observations indicate a very peculiar characteristic of BAT with respect to all other tissues that are reliant on a more reductive environment for their function^22^,^23^.

Glutathione (GSH) represents the major thiol-disulphide redox system within all cell types^24^ and changes in its concentration have been largely recognized to drive cell differentiation^25^. In recent years, many aspects of adipose tissue physiology have been demonstrated to tightly depend upon GSH redox system^26^,^27^,^28^. Adipocyte differentiation is boosted by conditions that promote intracellular compartment oxidation^29^. In particular, variations of GSH to GSSG ratio towards oxidizing conditions accompany adipogenesis^28^,^30^ and depletion of GSH in the course of differentiation accelerates white adipocyte maturation and increases triglyceride accumulation^28^. Interestingly, all conditions that have been reported to impinge BAT activation and/or WAT browning (*e.g.* acute physical exercise, cold exposure, inflammation and cancer) represent *per se* stressful hints that are associated with oxidative stress and could cause a remarkable shift of GSH redox state towards pro-oxidant conditions in plasma and tissues^14^,^31^,^32^. Despite this evidence, the role of GSH in the modulation of BAT activity and browning of WAT is completely unknown.

In the present report, we have considered the possibility that the decrease of GSH concentration could drive the browning process. We have demonstrated that GSH levels are reduced during white adipose cells browning and that treatment with the non-toxic inhibitor of GSH synthesis buthionine sulfoximine (BSO) elicits the up-regulation of genes related to uncoupling respiration (*e.g.* Ucp1 and Ppargc1a) in brown and white adipocytes. Importantly, we also found that the induction of the redox sensitive transcription factor FoxO1 is essential for the browning response.

## Results

### Activation of the thermogenic program is associated with GSH decrement

**Figure 1:**
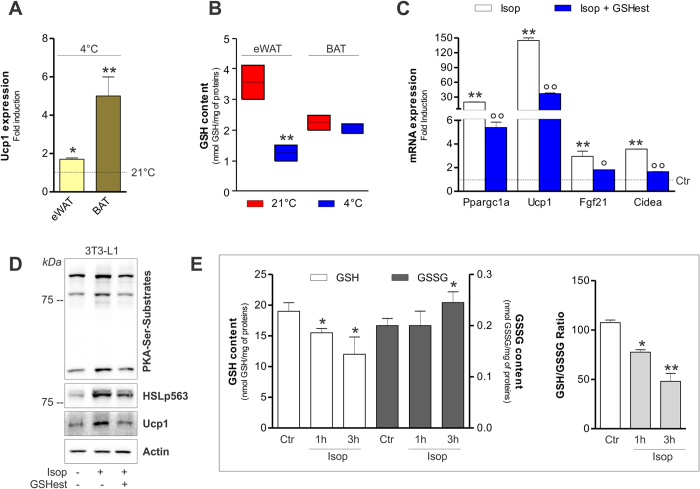
GSH decrease is associated with the induction of thermogenic program in adipose tissues. (**A**) mRNA levels of Ucp1 were measured through RT-qPCR in eWAT and BAT from mice exposed to cold (4 °C for 20 h). Data are reported as fold induction with respect to control mice (21 °C) and expressed as means ± S.D. (n = 3 mice per group; *p < 0.01, **p < 0.001 vs 21 °C). (**B**) GSH content was measured by HPLC in mouse eWAT and BAT. Data are reported as nmol GSH/mg protein and expressed as means ± S.D. (n = 3 mice per group, **p < 0.001 vs 21 °C). (**C**) mRNA levels of Ppargc1a, Ucp1, Fgf21 and Cidea were measured through RT-qPCR in 3T3-L1 adipocytes exposed to 1 h isoproterenol treatment. GSH ester (5 mM) was added 30 min prior isoproterenol addition and maintained throughout the experiment. Data are reported as fold induction with respect to control and expressed as means ± S.D. (n = 4; **p < 0.001 vs Ctr; °p < 0.01, °°p < 0.001 vs isoproterenol). (**D**) Protein levels of PKA-Ser-substrates, phospho-active HSL (HSLp563) and Ucp1 were measured by Western blot in 3T3-L1 adipocytes treated as described in (**C**). Actin was used as loading control. The full-length original blots are reported in Suppl. Fig. S2. (**E**) GSH and GSSG content was measured by HPLC in 3T3-L1 adipocytes exposed to isoproterenol treatment. Data are reported as nmol GSH or GSSG/mg proteins (*left panel*) or as GSH/GSSG ratio (*right panel*) and expressed as means ± S.D. (n = 4; *p < 0.01, **p < 0.001 vs Ctr).

Intracellular GSH varies during differentiation process in many cell types^33^,^34^. In particular, we previously showed that during differentiation, 3T3-L1 cells shift towards an oxidative milieu, which is functional in determining the induction of adipogenic genes including C/EBPβ^28^, a transcription factor also linked to adipocyte white-to-brown conversion^35^. Whether changes in redox state also drive trans-differentiation in white adipocytes has been poorly investigated. Cold is a physiological stimulus that has been demonstrated to induce WAT browning^36^. This prompted us to analyse the effects of an acute cold exposure on GSH content in epididymal WAT (eWAT). To this end, we exposed 6 weeks old mice to cold (4 °C) for 20 h and we analyzed the expression of Ucp1, a well-accepted marker of thermogenesis activation. As shown in Fig. 1A, Ucp1 was up-regulated both in eWAT and BAT, indicating that such treatment effectively stimulates a thermogenic program in adipose depots. Interestingly, a marked reduction of GSH content was determined upon cold exposure in eWAT (Fig. 1B), whereas, no significant variations were observed in BAT (Fig. 1B). However, under basal condition, we detected a lower GSH amount in BAT (about 2 nmol/mg proteins) with respect to eWAT (about 4 nmol/mg proteins) that could be indicative of a higher basal oxidative environment in brown adipose cells (Fig. 1B). The value of GSSG concentration was under the limit of detection under our experimental conditions in mouse adipose depots. To further associate changes of GSH content with the trans-differentiation process we treated 3T3-L1 white adipocytes with the β_3_-adrenergic agonist isoproterenol, a drug that is known to induce the white-to-brown conversion in cultured adipocytes^37^,^38^. Expectedly, isoproterenol triggered the up-regulation of the genes related to brown adipose cells phenotype (i.e. Ppargc1a, Ucp1, Fgf21, Cidea) (Fig. 1C) and increased phosphorylation of PKA-serine-substrates as well as phosphorylation of HSL at Ser563 (Fig. 1D). In parallel, we noticed a rapid and progressive reduction of GSH levels concomitant to a decrease of GSH/GSSG ratio (Fig. 1E). To assess whether GSH lowering was causally involved in the induction of the brown-like changes, we treated white adipocytes with GSH ester 30 min prior to isoproterenol addition. GSH ester significantly restrained the induction of the brown-related mRNAs (Fig. 1C) as well as cAMP-PKA signal transduction pathway (Fig. 1D).

### GSH depletion through BSO treatment selectively affects adipose tissue and elicits its shape remodelling

**Figure 2:**
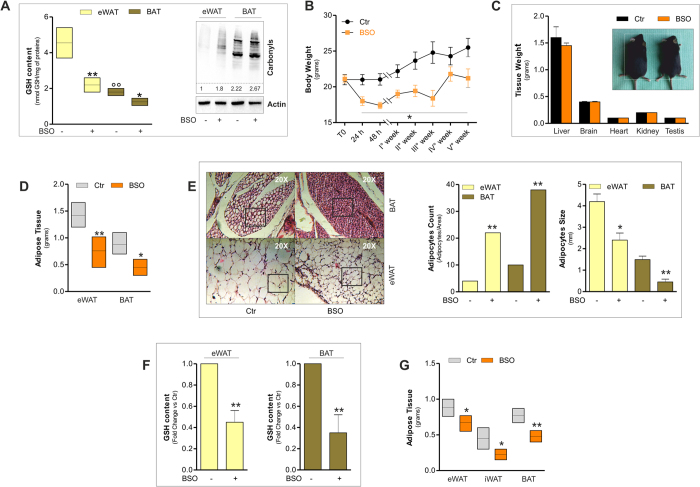
Glutathione depletion by BSO induces fat remodelling in mice. (**A**) GSH content was measured by HPLC in eWAT and BAT from mice treated with BSO (20 mM in drinking water) for 5 weeks (*left panel*). Protein oxidation was determined by assaying carbonyl residues through Western blot analysis (*right panel*). Actin was used as loading control. Below are reported the densitometric analyses of the immunoreactive bands normalized to actin. The full-length original blots are reported in Suppl. Fig. S3. Data are reported as nmol GSH/mg proteins and expressed as means ± S.D. (n = 4 mice per group, *p < 0.01, **p < 0.001 vs Ctr; °°p < 0.001 vs eWAT Ctr). (**B**) Body weights of mice treated with BSO (20 mM in drinking water) up to 5 weeks. Data are expressed as means ± S.D. (n = 4 mice per group; *p < 0.01 vs Ctr). (**C**) Tissue weights of mice treated with BSO as described in (**A**). Data are expressed as means ± S.D. (n = 4 mice per group). Representative photographs of C57/BL6 mice are reported in the inset. (**D**) Weights of eWAT and BAT from mice treated with BSO as described in (**A**). Data are expressed as means ± S.D. (n = 4 mice per group; *p < 0.01, **p < 0.001 vs Ctr). (**E**) H&E histological analysis (*left panel*) with relative adipocytes count per area and size (*right panels*) of eWAT and BAT from mice treated with BSO as described in (**A**). Data are expressed as means ± S.D. (n = 4 mice per group; *p < 0.01, **p < 0.001 vs Ctr). (**F**) GSH content of eWAT and BAT from mice treated with BSO for 24 h (20 mM in drinking water). Data are reported as fold change with respect to control and expressed as means ± S.D. (n = 4 mice per group; **p < 0.001 vs Ctr). (**G**) Weights of eWAT, iWAT and BAT from mice treated with BSO as described in (**F**). Data are expressed as means ± S.D. (n = 4 mice per group; *p < 0.01, **p < 0.001 vs Ctr).

On the basis of these observations, we asked whether GSH depletion through the chemical inhibition of its synthesis could induce the browning program in mouse WAT. To this end, we treated mice by adding BSO in drinking water (20 mM) up to 5 weeks. GSH content in eWAT was significantly lowered of about 50% in BSO-treated mice reaching a value similar to that observed in untreated BAT. Moreover, BSO was able to efficiently induce GSH depletion also in BAT (Fig. 2A). The GSH decrement was associated with an accumulation of oxidized proteins both in terms of carbonyls (Fig. 2A) and 4-hydroxynonenal (4-HNE) adducts (Suppl. Fig. S1A) in eWAT. Remarkably, in line with the lower GSH content, the level of oxidized proteins was higher in BAT with respect to eWAT under basal conditions (Fig. 2A, Suppl. Fig. S1A), confirming a higher oxidative environment in BAT. However, BSO treatment only slightly increased protein oxidation in BAT, as evidenced by minor increase of protein carbonyls with respect to eWAT and not significant changes in the levels of 4-HNE protein adducts (Fig. 2A, Suppl. Fig. S1A).

During BSO treatment mice did not show any variation in water (data not shown) and food consumption (Suppl. Fig. S1B). Notwithstanding, BSO-treated mice rapidly underwent weight loss (15% reduction at 24 h). Similar slimming effects were observed in mice exposed to cold for 20 h (10% weight reduction) (Suppl. Fig. S1C). BSO-treated mice remained lighter with respect to untreated mice up to the end of treatment (5 weeks) (Fig. 2B). At 5 weeks, the weights of liver, brain, heart, kidney and testis of BSO-treated mice were not significantly different from controls (Fig. 2C). The slimming effects of BSO were associated with the diminution of adipose depots mass. Actually, eWAT and BAT weights were significantly reduced upon 5 weeks of BSO treatment (Fig. 2D). In particular, histological analyses showed that BSO caused a profound tissue shape remodelling both in eWAT and BAT consisting in a reduced lipid droplet size as well as increased number of adipocytes per area (Fig. 2E).

### BSO treatment induces adipose cells browning and mitochondrial uncoupling via FoxO1

**Figure 3:**
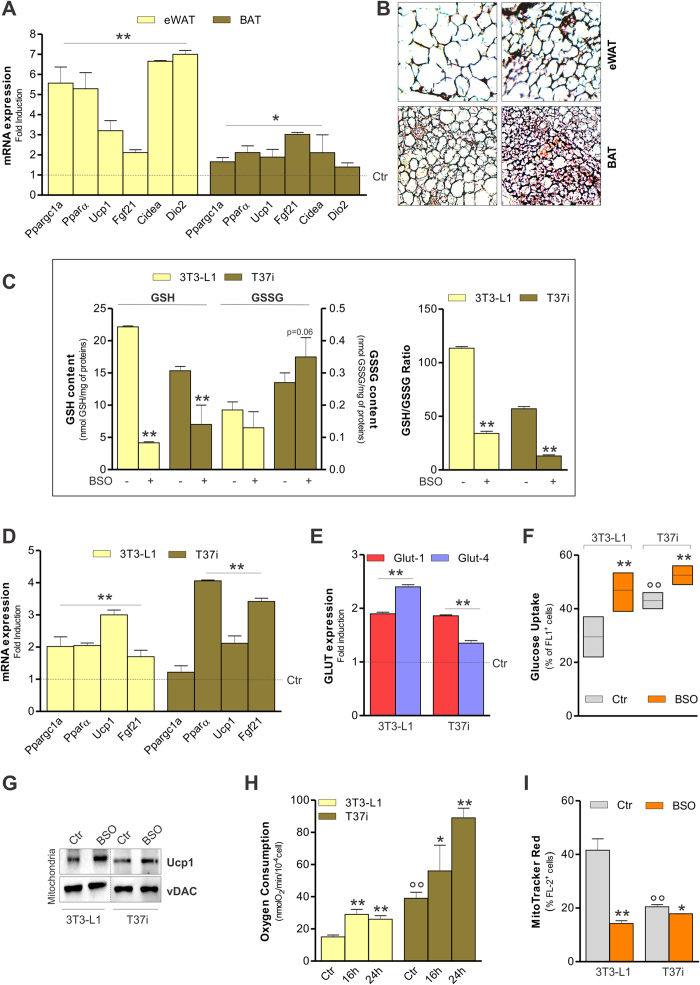
BSO treatment activates thermogenic program in adipose tissue. (**A**) mRNA levels of Ppargc1a, Pparα, Ucp1, Fgf21, Cidea and Dio2 measured in adipose tissue of mice treated with BSO for 24 h (20 mM in drinking water). Data are reported as fold induction with respect to control and expressed as means ± S.D. (n = 4 mice per group; *p < 0.05, **p < 0.001 vs Ctr). (**B**) Immunohistochemical analysis of Ucp1 protein in eWAT and BAT from mice treated as in (**A**). (**C**) GSH and GSSG content in 3T3-L1 and T37i adipocytes treated with BSO (1 mM for 24 h). Data are reported as nmol GSH or GSSG/mg proteins (*left panel*) or as GSH/GSSG ratio (*right panel*) and expressed as means ± S.D. (n = 4; **p < 0.001 vs Ctr). (**D**) mRNA levels of Ppargc1a, Pparα, Ucp1 and Fgf21 measured in 3T3-L1 and T37i adipocytes treated as in (**C**). Data are reported as in (A) (n = 4; **p < 0.001 vs Ctr). (**E**) mRNA levels of Glut-1 and Glut-4 measured in 3T3-L1 and T37i adipocytes treated as in (**C**). Data are reported as in (**A**) (n = 4; **p < 0.001). (**F**) Glucose uptake in 3T3-L1 and T37i adipocytes treated as in (**C**). Data are reported as percentage of FL-1 positive cells and are expressed as means ± S.D. (n = 4; **p < 0.001 vs Ctr; °°p < 0.001 vs 3T3-L1 Ctr). (**G**) Western blot analysis of Ucp1 in crude mitochondria derived from 3T3-L1 and T37i adipocytes treated as in (**C**). vDAC was used as loading control. The full-length original blots are reported in Suppl. Fig. S4. (**H**) Mitochondrial oxygen consumption measured through a polarograhic method in 3T3-L1 and T37i adipocytes treated with BSO (1 mM for 16 and 24 h). Data are reported as nmolO_2_/min/10^−4^ cells and expressed as means ± S.D. (n = 4; *p < 0.05, **p < 0.001 vs Ctr; °°p < 0.001 vs 3T3-L1 Ctr). (**I**) Mitochondrial membrane potential measured by cytofluorimetric analysis after MitoTracker Red staining in 3T3-L1 and T37i adipocytes treated as in (**C**). Data are reported as percentage of FL-2 positive cells and expressed as means ± S.D. (n = 4; *p < 0.05, **p < 0.001 vs Ctr; °°p < 0.001 vs 3T3-L1 untreated).

Given that GSH synthesis inhibition was able to rapidly trigger weight loss in mice, we moved at analysing the effect of BSO at 24 h treatment. At this time, BSO led to a significant GSH reduction both in eWAT and BAT (Fig. 2F) that was associated with morphological changes as well as weight loss in all adipose depots (Fig. 2G, Suppl. Fig. S1D). To investigate whether BSO could elicit the browning program in eWAT and activation of BAT, we analysed the expression of genes related to thermogenesis. As reported in Fig. 3A, mRNA level of Ppargc1a, Pparα, Ucp1, Fgf21, Cidea and Dio2 were significantly up-regulated in eWAT of mice treated with BSO for 24 h. Although at lesser extent, the expression of Ppargc1a, Pparα, Ucp1, Fgf21 and Cidea was up-regulated in BAT as well. Immunohistochemical analyses confirmed the shift of eWAT towards a brown-like phenotype. Indeed, increased level of Ucp1 was detected in eWAT of BSO-treated mice (Fig. 3B). Ucp1 protein increased in BAT as well, implying the activation of the thermogenic program upon BSO treatment (Fig. 3B).

To confirm the data obtained *in vivo* and to more deeply investigate the mechanisms underlying the WAT browning and BAT activation, we treated 3T3-L1 white and T37i brown adipocytes with BSO for 24 h. At this time, GSH and GSH/GSSG ratio were markedly decreased both in 3T3-L1 and T37i cells (Fig. 3C). A lower amount of glutathionylated proteins was also observed (Suppl. Fig. S1E). Indicators of brown phenotype including Ppargc1a, Pparα, Ucp1 and Fgf21 were up-regulated in these cells (Fig. 3D). Other brown adipocyte hallmarks, such as enhanced level of Glut-1 and Glut-4 expression, were also evidenced (Fig. 3E). As consequence, glucose uptake was more efficient in BSO-treated with respect to untreated 3T3-L1 and T37i adipocytes (Fig. 3F). The effectiveness of mitochondrial uncoupling activity was assayed by analysing the mitochondrial level of Ucp1, oxygen consumption and mitochondria depolarization. Western blot analysis performed on crude mitochondrial proteins revealed an augmentation of Ucp1 content in BSO-treated adipocytes (Fig. 3G). BSO efficiently increased oxygen consumption (Fig. 3H) and caused the drop of mitochondrial membrane potential both in T37i and 3T3-L1 cells (Fig. 3I), with T37i having the lower degree of variations. In line with their brown nature, under basal condition T37i cells displayed higher values of glucose uptake and oxygen consumption (Fig. 3F,H). Coherently, T37i also had lower mitochondrial trans-membrane potential compared to 3T3-L1 adipocytes (Fig. 3I).

**Figure 4:**
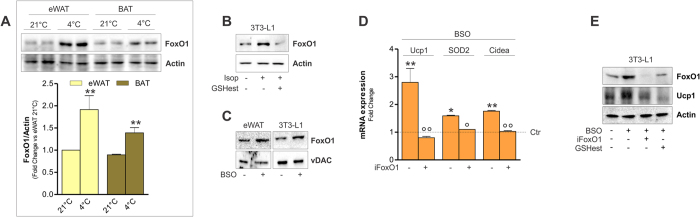
BSO-mediated Ucp1 induction is downstream of FoxO1. (**A**) FoxO1 levels were detected by Western blot in eWAT and BAT from mice exposed to cold (4 °C for 20 h). Below are reported the densitometric analyses of the immunoreactive bands normalized to actin. The full-length original blots are reported in Suppl. Fig. S5. Data are reported as fold change with respect to eWAT 21 °C and expressed as means ± S.D. (n = 3 mice per group, **p < 0.01 vs 21 °C). (**B**) FoxO1 levels were detected by Western blot in 3T3-L1 adipocytes exposed to 1 h isoproterenol treatment. GSH ester (5 mM) was added 30 min prior isoproterenol addition and maintained throughout the experiment. Actin was used as loading control. The full-length original blots are reported in Suppl. Fig. S6. (**C**) FoxO1 levels were detected by Western blot in eWAT from mice treated with BSO (20 mM in drinking water for 24 h) or 3T3-L1 adipocytes treated with BSO (1 mM for 24 h). vDAC was used as loading control. The full-length original blots are reported in Suppl. Fig. S7. (**D**) Ucp1, SOD2 and Cidea mRNAs were detected by RT-qPCR in 3T3-L1 adipocytes down-regulating FoxO1 (iFoxO1) and treated with BSO as described in (**C**). Data are reported as fold induction with respect to control and expressed as means ± S.D. (n = 4; *p < 0.05, **p < 0.001 vs Ctr). (**E**) Ucp1 levels were detected by Western blot in 3T3-L1 adipocytes down-regulating FoxO1 (iFoxO1) and treated with BSO as described in (****C****). GSH ester (5 mM) was added 30 min prior BSO addition and maintained throughout the experiment. The full-length original blots are reported in Suppl. Fig. S8.

Many transcription factors are involved in the expression of Ucp1 among which FoxO1 is included^39^. We previously showed that FoxO1 governs the expression of lipid oxidative genes in white adipose cells via a redox-dependent manner^3^,^40^. Interestingly, the thermogenic equipment mounted by cold exposure in mice and isoproterenol treatment in 3T3-L1 cells was also characterized by the induction of FoxO1 (Fig. 4A,B). Pre-treatment with GSH ester, which we showed to significantly blunt adipocyte browning, was able to buffer FoxO1 up-regulation in 3T3-L1 adipocytes (Fig. 4B). Remarkably, GSH lowering through BSO treatment was able to efficiently up-regulate FoxO1 levels both in WAT and 3T3-L1 adipocytes mimicking what observed during cold and isoproterenol treatment respectively (Fig. 4C).

We next asked whether the BSO-mediated induction of the browning program was managed by FoxO1. To this end, we transfected 3T3-L1 adipocytes with FoxO1 siRNA prior to BSO treatment. As reported in Fig. 4D, the up-regulation of brown-like markers (i.e. Ucp1, SOD2 and Cidea) induced by BSO was significantly blunted in 3T3-L1 cells down-regulating FoxO1. Moreover, pre-treatment with GSH ester was able to inhibit the increase of FoxO1 as well as the level of Ucp1 protein (Fig. 4E), pointing to a GSH-dependent modulation of the thermogenic program.

## Discussion

Physical exercise and cold exposure represent stressful conditions leading to increased energy dissipation through WAT browning and BAT activation^8^,^9^,^10^,^11^. Furthermore, such conditions generate alterations of redox homeostasis leading to pro-oxidant states^14^,^31^,^32^. Thiol-based reactions have been widely implicated in the control of many cellular processes including cell differentiation^33^,^34^.

Here we have reported that a decrease of GSH accompanies BAT activation and WAT browning and that the modulation of GSH levels by BSO could be an effective tool to drive trans-differentiation or metabolic adaptation in adipose cells. The slimming effects of BSO are known since many years^41^,^42^,^43^,^44^. However, the molecular mechanism(s) by which GSH regulates body fat content are currently not fully established. Up to date, one of the hypotheses given to explain the effects of BSO in WAT remodeling was that fat precursor cells could have diminished replicative potential due to the reduction of telomere length^41^. Alternatively, it was proposed that WAT undergoes atrophy as consequence of diminished tissue viability^43^. More conceivable was the idea that reduction of WAT mass was due to increased locomotor activity accompanied by enhanced energy expenditure^44^. The intriguing molecular explanation of such phenomenon was that BSO is able to induce the expression of Ucp2 and Ucp3 resulting in enhanced uncoupling respiration and energy dissipation in WAT. Nevertheless, no effect either on Ucp1 expression in WAT or in BAT activity have been reported yet. In this work we show that pharmacological GSH depletion by BSO builds-up an intense metabolic rearrangement in WAT and BAT that culminates in a significant fat mass reduction. A similar body weight loss was observed in mice exposed to cold even under *ad libitum* food intake, suggesting that BSO triggers a “physiological” affordable change at least for this animal species. BSO treatment promotes Ucp1-mediated thermogenic program in white and brown adipose cells. Many are the proofs we provided indicating that the expression of thermogenesis-related genes is inversely correlated with GSH concentration. We have shown that BAT has a more oxidized redox state with respect to WAT and becomes more active when GSH synthesis is inhibited. By stimulating the white-to-brown conversion hormonally, we observed that a rapid drop of GSH concentration and decreased GSH/GSSG ratio accompany the up-regulation of brown-related genes and that such an event is significantly blunted by supplementing adipocytes with GSH ester. Another confirmation derives from *in vivo* experiments in which we illustrate that brown-like metabolic remodeling of mouse eWAT upon cold exposure is accompanied by a significant reduction of GSH. More excitingly, here we show that by forcing GSH redox state towards the similar pro-oxidant conditions of BAT, the expression of Ucp1 and other genes related to uncoupling respiration is enhanced in white adipose cells and tissue. Specifically, Ucp1 mRNA is strongly up-regulated and this results in the drop of mitochondrial trans-membrane potential and higher degree of oxygen consumption. The shift towards a brown-like metabolism is also confirmed by the induced expression of Glut genes and increased capacity of white adipocytes to incorporate glucose. Of notice, cold exposure does not trigger the decrement of GSH in BAT, implying that the high oxidative environment of this tissue under basal conditions is already permissive towards the induction of the thermogenic program.

Also genetic manipulation of GSH concentration has been found to cause changes in body mass composition^45^. A diminished percentage of body fat without any changes in the lean mass was observed in mice having global ablation of the modifier subunit of GCL (GCLM), which is responsible for the modulation of GCL catalytic activity^45^. In particular, even though preserving 80% GSH content compared to wild type animals, these mice had a more oxidized thiol redox state, resulted lighter and highly resistant to weight gain and alteration of metabolic parameters during high fat diet. Accordingly, we have observed that even though mice normally growth upon BSO treatment they remained slimmer than control up to five weeks treatment, suggesting that energy intake is not exceeding to accumulate fats. Increased oxygen consumption was also observed, however, any investigation was made regarding the possible contribution of uncoupling respiration on such increased mitochondrial activity. Overlapping metabolic profile was observed by Findeisen and co-workers on mice treated with BSO that resulted highly resistant to high fat diet-induced obesity as well^44^. Collectively these and our findings give effort to the idea that the metabolic adaptations triggered by BSO are strictly dependent on GSH lowering and not to unspecific effects of this GSH synthesis inhibitor.

FoxO1 is a redox-sensitive transcription factors that finely modulates lipid metabolism in adipose cells^1^. Among the roles of FoxO1 in adipose tissue metabolism the induction of Ucp1 protein is also included^39^. In line with this, we have substantiated that the expression of brown-related genes such as Ucp1 is modulated by BSO through a FoxO1 pathway in white adipocytes. Indeed, GSH ester supplementation efficiently restrains both FoxO1 activation and the expression of Ucp1 gene.

In recent years, igniting thermogenesis within BAT and WAT has drawn the attention of researchers working in the field of obesity and its related diseases^46^. Physiological stimuli (i.e. physical exercise, cold, fasting) have been found to increase body energy dissipation via the action of hormones, such as orexin, irisin and Fgf21^8^,^9^,^47^. The discovery of these hormones as “burning” factors raised expectations of their therapeutic potential to prevent fat gain and treat metabolic diseases^48^,^49^,^50^. However, since many of the beneficial actions have been studied in mice, the scientific community is still critical against the possible therapeutic use of these hormones. For instance, the function of irisin is still unclear and controversial in humans and the use of irisin as therapeutic tool has been recently criticised^51^,^52^,^53^. Moreover, our data support the idea that, to be successful, the hormone-dependent thermogenic cascade must proceed through an oxidative intracellular environment. Actually, in the presence of GSH ester, isoproterenol is ineffective in triggering either the activation of PKA and its downstream effector (i.e. HSL) or the thermogenic program. Intriguingly, it was reported that obese rats have higher GSH content in white adipose depots^27^. With this in mind, we can postulate that the simple treatment with a “thermogenic” hormone may be not sufficient to induce the browning program if an adequate drop of GSH content is not reached. Notably, the cold-induced white-to-brown conversion was demonstrated to arise also when the adrenergic cascade is blocked, highlighting that white adipocytes directly sense temperature variations^54^. Thus, it is possible to postulate that cold directly causes GSH redox imbalance and this event is the genuine inducer of the expression of uncoupling genes in a way that could be independent of hormonal stimulation. Conceivably, the redox imbalance caused by GSH decrease may also act as an enhancer of hormonal lipolytic cascade resulting in a more efficient dissipation of stored energy.

Overall our findings emphasize the cardinal role of GSH redox system in metabolic adaptation of adipocytes through enhanced energy dissipation. Phase I clinical studies with continuous-infusion of BSO have shown that GSH can be depleted without undue normal tissue toxicity^55^,^56^. Therefore by virtue of its ability to dissipate excessive energy through mitochondrial uncoupling both in WAT and BAT, BSO may be considered for future studies as a drug with strong therapeutic potential for metabolic diseases treatment.

## Methods

### Cell lines, treatments and transfections

3T3-L1 murine adipocytes (American Type Culture Collection, Bethesda, MD, USA) were cultured and differentiated as previously described^3^. All experiments were performed in fully differentiated (day 8) 3T3-L1 white adipocytes. T37i murine cell line was kindly provided by Prof. Marc Lombes (Inserm U693, Paris, France) and was grown and differentiated as described by Nakae *et al*.^57^ with some modifications. Briefly, cells were grown in DMEM/F-12 supplemented with 10% fetal calf serum until confluence. Two days later, the cells were treated with differentiation medium (DMEM containing 10% fetal bovine serum, 0.5 mM 3-isobutyl-1-methylxanthine, 1 μM dexamethasone, 1 μg/mL insulin, 1 μM rosiglitazone and 2 nM triiodothyronine). The maintenance medium (DMEM supplemented with 10% fetal bovine serum, 1 μM rosiglitazone and 2 nM triiodothyronine) was changed every 48 h and all experiments were performed after 8 days of differentiation. All experiments were performed in fully differentiated (day 8) T37i brown adipocytes. BSO (Sigma-Aldrich, St. Louis, MO, USA) was dissolved in PBS and added in culture medium at a final concentration of 1 mM and maintained throughout the experiment. GSH ester (Sigma-Aldrich) was dissolved in culture medium at a concentration of 5 mM and maintained throughout the experiment. Isoproterenol hydrochloride (Sigma-Aldrich) was dissolved in PBS and added in culture medium at a final concentration of 10 μM and maintained throughout the experiment. Fully differentiated adipocytes were transfected with a siRNA duplex directed against the mouse FoxO1 (Santa Cruz Biotechnologies, Santa Cruz, CA, USA) sequence. Transfection with a scramble siRNA duplex (scr), with no homology to other mouse mRNA, was used as control. Thirty hours after transfection cells were treated with BSO.

### Mice and treatments

We conducted all mouse experimentations in accordance with accepted standard of human animal care and with the approval by relevant national (Ministry of Welfare) and local (Institutional Animal Care and Use Commettee, Tor Vergata University) committees. Sixteen C57BL/6 adult (1 months-age-old) male mice (purchased from Harlan Laboratories S.r.l., Urbino, Italy) were randomly divided in 2 groups: untreated mice (control group, n = 8 mice) or BSO-treated mice (BSO, n = 8 mice). BSO-treated mice were randomly divided in two subgroups: 24-hours-treated mice (n = 4 mice) or 5-weeks-treated mice (n = 4 mice). BSO was supplied in drinking water at the final concentration of 20 mM. Six C57BL/6 (6 weeks-age-old) male mice were randomly divided in two groups: room temperature group (control group, 21 °C) or cold exposed group (4 °C). Cold exposure was maintained for 20 hours. All mice were housed with 12 h light/dark cycles and had free food and water access. After cervical dislocation, tissues were explanted and immediately processed.

### Isolation of mitochondria

Crude mitochondria from adipose tissue were obtained as described by Wieckowski *et al*.^58^. Purified mitochondria from 3T3-L1 and T37i were obtained according to Kristian *et al*.^59^ with some modifications. Briefly, cells were lysed at 4 °C in a homogenization buffer (210 mM mannitol, 70 mM sucrose, 5 mM HEPES, pH 7.4) supplemented with EGTA (Sigma-Aldrich), albumin 0.5% and protease and phosphatase inhibitors (Sigma-Aldrich) and centrifuged at 600 × *g* for 3 min, 4 °C to pellet nuclei and heavy particles. The supernatant was collected and centrifuged at 17000 × *g* for 17 min, 4 °C to pellet mitochondria that were then washed 2 times by centrifugation at 17000 × *g* for 15 min, 4 °C.

### Determination of protein carbonylation

Carbonylated proteins were detected using the Oxyblot Kit (Merck Millipore, Darmstadt, Germany) as previously described^3^. Briefly, 15 μg of proteins were reacted with 2,4 dinitrophenylhydrazine (DNP) for 15 minutes at 25 °C. Samples were resolved on 10% SDS-polyacrylamide gels and DNP-derivatized proteins were identified by Western blot analysis using an anti-DNP antibody and an appropriate horseradish peroxidase-conjugated secondary antibody.

### Gel electrophoresis and Western blot

Crude mitochondria, cell pellets, eWAT and BAT were lysed in RIPA buffer (50 mM Tris-HCl, pH 8.0, 150 mM NaCl, 12 mM deoxycholic acid, 0.5% Nonidet P-40 and protease and phosphatase inhibitors). Protein samples were used for SDS-PAGE followed by Western blotting. Nitrocellulose membranes were stained with primary antibodies against β-Actin, FoxO1 (Santa Cruz Biotechnologies), vDAC, Ucp1 (Abcam, Cambridge, UK), pHSL, PKA-Ser-Substrates (Cell Signaling Technologies, Danver, MA, USA), GSH (Enzo Lifescience, Farmingdale, NY, USA) and 4-hydroxy-2-nonenal (4-HNE) histidine (gently donated by Prof. Uchida, School of Bioagricultural Sciences, Nagoya University, Japan) all diluted 1:1000. Afterward, the membranes were incubated with the appropriate horseradish peroxidase-conjugated secondary antibody, and immunoreactive bands were detected by a Fluorchem Imaging System upon staining with ECL Selected Western Blotting Detection Reagent (GE Healthcare, Pittsburgh, PA, USA). Immunoblots reported in the figures are representative of at least three experiments that gave similar results.

### RT-qPCR analysis

Total RNA was extracted using TRI Reagent^®^ (Sigma-Aldrich). Three μg of RNA was used for retro-transcription with M-MLV (Promega, Madison, WI). qPCR was performed in triplicates by using validated qPCR primers (BLAST), Ex TAq qPCR Premix, and the Real-Time PCR LightCycler II (Roche Diagnostics, Indianapolis, IN) as previously described^40^. mRNA levels were normalized to actin mRNA, and the relative mRNA levels were determined by using the 2^−ΔΔCt^ method.

### Determination of GSH levels

Intracellular GSH and GSSG was assayed upon formation of S-carboxymethyl derivates of free thiols with iodoacetic acid, followed by the conversion of free amino groups to 2,4-dinitrophenyl derivative by the reaction with 1-fluoro-2,4 dinitrobenzene as previously described^60^.

### Assay of mitochondrial membrane potential and glucose uptake

For evaluation of mitochondrial transmembrane potential cells were incubated with Mitotracker Red (Life Technologies Ltd) at concentration of 200 nM (30 min, 37 °C) prior to cytofluorimetric analysis. For measurement of glucose uptake cells were incubated with the fluorescent glucose analog 2-NBDG (Life technologies Ltd) at concentration of 100 μM (30 min, 37 °C) prior to cytofluorimetric analysis.

### Immunohistochemistry

Adipose tissues were explanted and immediately fixed overnight in formalin solution (Sigma-Aldrich), washed in water, dehydrated and embedded in paraffin. Paraffin-embedded tissues were cut in 7 μm sections and stained with haematoxylin and eosin (H&E) or with Ucp1 antibody. The images reported are representative of one experiment out of four that gave similar results. Adipocytes size and number per area were calculated by using an ImageJ plugin (Adipocytes Tool).

### Oxygen consumption

Oxygen consumption rate was determined in 3T3-L1 white and T37i brown adipocytes, using the Oxygraph Plus oxygen electrode system (Hansatech Instruments Ltd, Norfolk, UK). At the end of differentiation, cells were treated with BSO for 24 h, collected and resuspended with complete culture medium in the oxygraph chamber. Real-time oxygen consumption was recorded at 28 °C, for 5 min.

### Statistical analysis

The results are presented as mean ± S.D. Statistical evaluation was conducted by ANOVA followed by the post Student-Newman-Keuls. Differences were considered to be significant at P < 0.05.

## Additional Information

**How to cite this article**: Lettieri Barbato, D. *et al.* Glutathione Decrement Drives Thermogenic Program In Adipose Cells. *Sci. Rep.* **5**, 13091; doi: 10.1038/srep13091 (2015).

## Supplementary Material

Supplementary Information
