# Supplementary material for: Glutathione Decrement Drives Thermogenic Program In Adipose Cells
Source: Sci Rep. 2015 Aug 11;5:13091. doi: 10.1038/srep13091 (PMC4531326; doi:10.1038/srep13091)
Supplement: Supplementary Information [file srep13091-s1.pdf]

Glutathione Decrement Drives Thermogenic Program In Adipose Cells

Daniele Lettieri Barbato [1]*, Giuseppe Tatulli[2]*, Stefano Maria Cannata[1], Sergio Bernardini[1], Katia Aquilano[1,2]*, Maria R. Ciriolo[1,2]*

[1]Dept. Biology, University of Rome "Tor Vergata", Via della Ricerca Scientifica 1, 00133 Rome, Italy

[2]Scientific Institute for Research Hospitalization and Health Care and Università Telematica San Raffaele Roma, Via di Val Cannuta 247, 00166 Rome, Italy

**These authors equally contributed to this work*

Address correspondence to:

Maria Rosa Ciriolo
Dept. Biology, University of Rome "Tor Vergata"
Via della Ricerca Scientifica 1
00133 Rome, Italy
ciriolo@bio.uniroma2.it

SUPPLEMENTARY MATERIAL

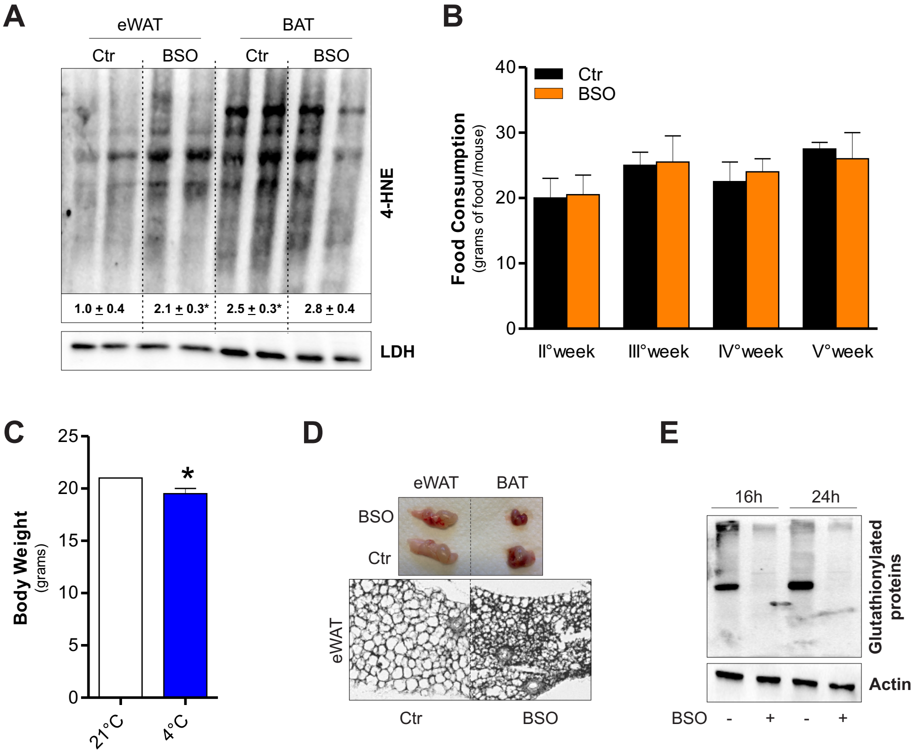

Supplementary Figure S1.

(**A**) Western blot analysis of 4-HNE protein adducts in eWAT and BAT from mice treated with BSO (20 mM in drinking water) for 24 h. Below are reported the densitometric analyses of the immunoreactive bands normalized to LDH and expressed as arbitrary units (*p<0.05 vs eWAT Ctr; n=4 mice per group).

(**B**) Means of weekly food consumption in mice treated with BSO (20 mM in drinking water) up to 5 weeks (n=4 mice per group).

(**C**) Means of body weight in mice exposed to cold (4°C) for 20 h (*p<0.01, n =4 mice per group).

(**D**) Representative photographs (*upper panel*) and H&E histological analyses (*bottom panel*) of eWAT and BAT from mice treated with BSO as described in (A).

(**E**) Western blot analysis of S-glutathionylated proteins by using a GSH antibody in 3T3-L1 cells treated with BSO (1 mM).

A B

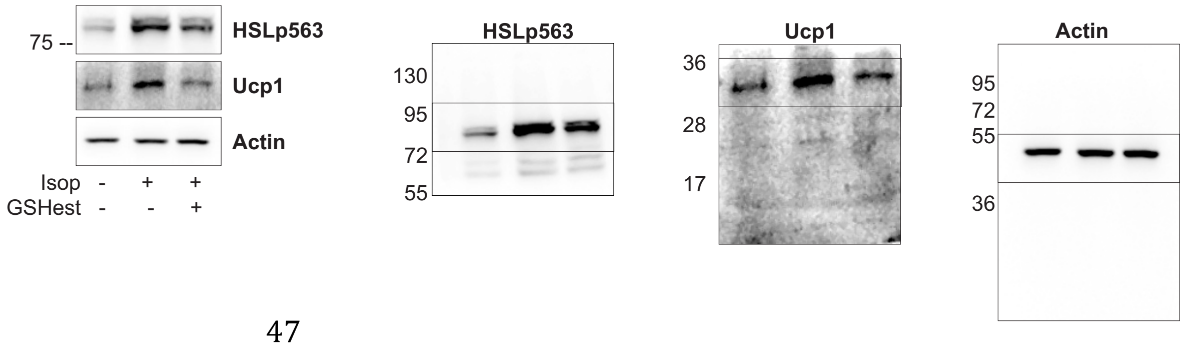

Supplementary Figure S2.

(**A**) Protein levels of phospho-active HSL (HSLp563) and Ucp1 were measured by Western blot in 3T3-L1 adipocytes. Actin was used as loading control.

(**B**) The cropped original blots are shown.

A B

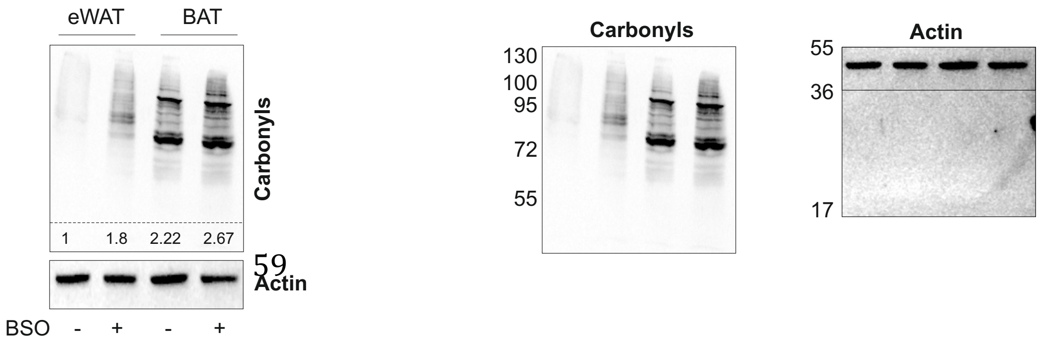

Supplementary Figure S3.

(**A**) Levels of protein oxidation determined by assaying carbonyl residues through Western blot analysis. Actin was used as loading control.

(**B**) The cropped original blots are shown.

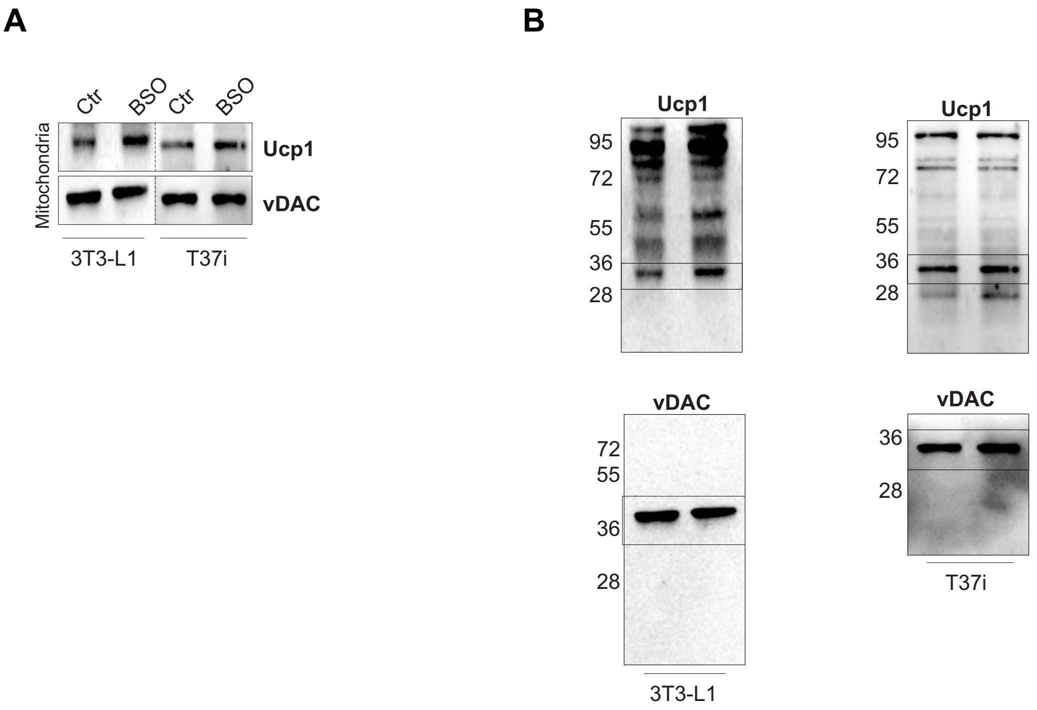

Supplementary Figure S4.

(**A**) Ucp1 levels was detected by Western blot analysis in crude mitochondria derived from 3T3-L1 and T37i adipocytes treated with BSO. vDAC was used as loading control.

(**B**) The cropped original blots are shown.

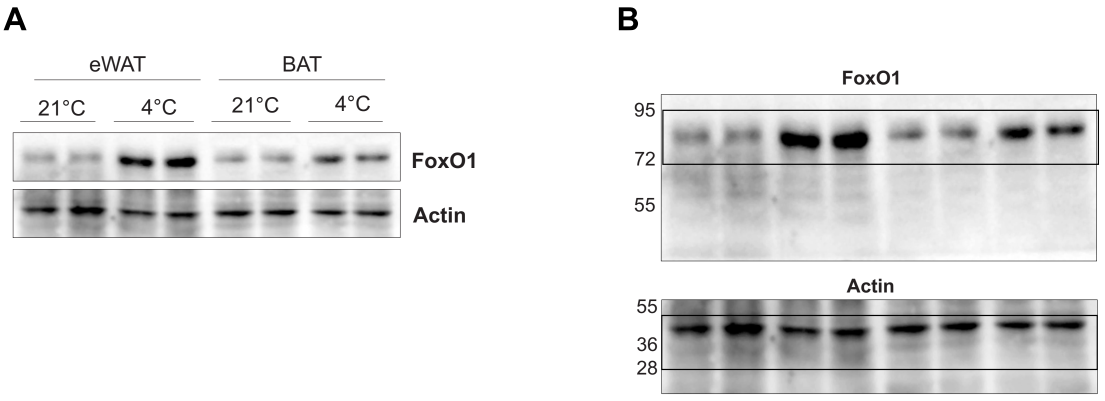

Supplementary Figure S5.

(**A**) FoxO1 levels were detected by Western blot in eWAT and BAT from mice exposed to cold (4 °C for 20 h). Actin was used as loading control.
(**B**) The cropped original blots are shown.

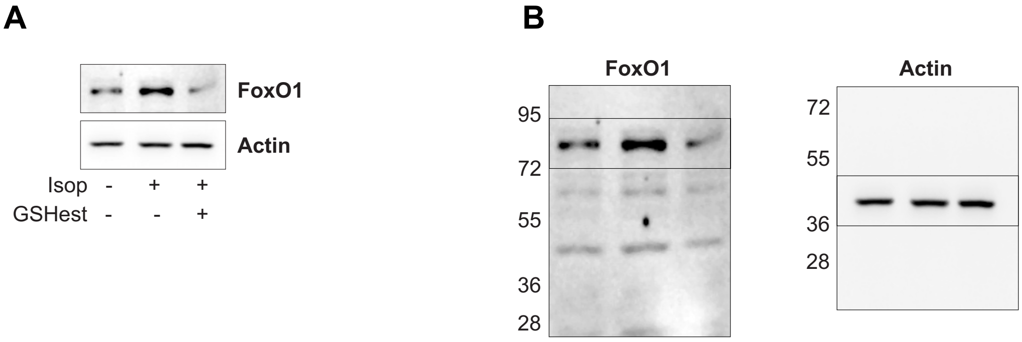

Supplementary Figure S6.
(**A**) FoxO1 levels were detected by Western blot in 3T3-L1 adipocytes exposed to 1 h isoproterenol treatment. GSH ester (5 mM) was added 30 min prior isoproterenol addition and maintained throughout the experiment. Actin was used as loading control.
(**B**) The cropped original blots are shown.

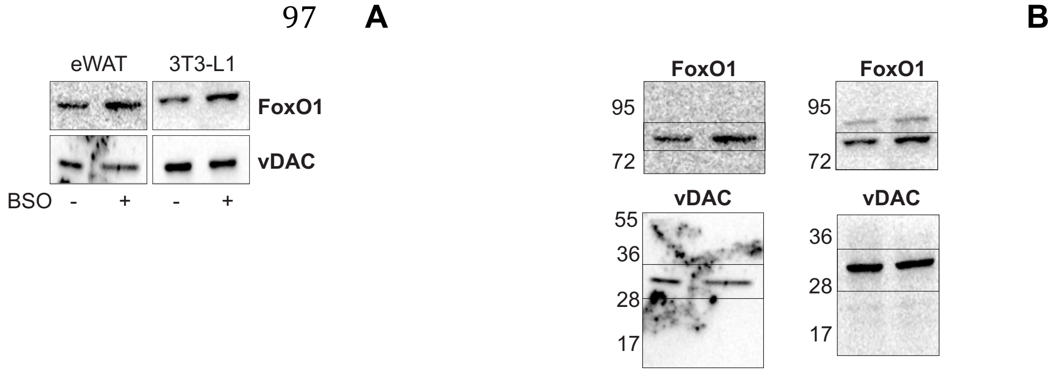

Supplementary Figure S7.

(**A**) FoxO1 levels were detected by Western blot in eWAT from mice treated with BSO (20 mM in drinking water) or 3T3-L1 adipocytes treated with BSO (1 mM) for 24 h. vDAC was used as loading control.

(**B**) The cropped original blots are shown.

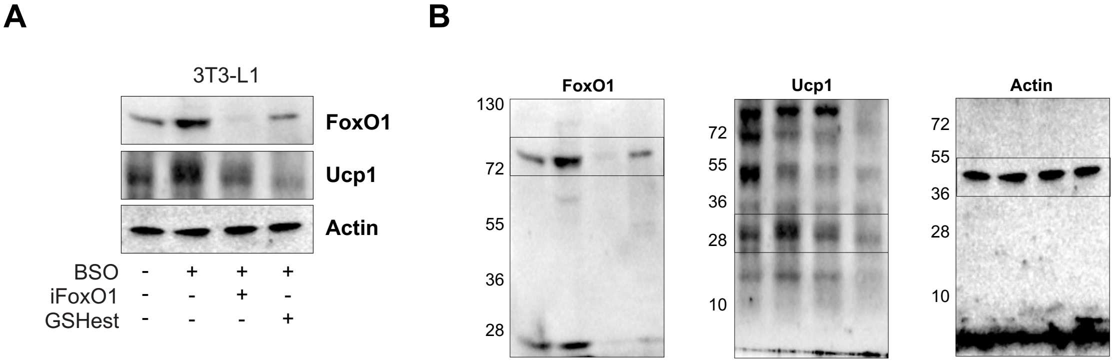

Supplementary Figure S8.

(**A**) Ucp1 levels were detected by Western blot in 3T3-L1 adipocytes down-regulating FoxO1 (iFoxO1) and treated with BSO. GSH ester (5 mM) was added 30 min prior BSO addition and maintained throughout the experiment.

(**B**) The cropped original blots are shown.
